# Prediction of liver stiffness by serum indexes in HCV-infected patients with or without HIV coinfection

**DOI:** 10.1097/MD.0000000000027838

**Published:** 2021-11-19

**Authors:** Nicolás Merchante, Álvaro Mena, Juan-Manuel Pascasio, Andrés Marco, Manuel Rodriguez, Manuel Hernandez-Guerra, Miguel-Angel Simón

**Affiliations:** a Unit of Infectious Diseases and Microbiology, Valme University Hospital, Sevilla, Spain; b Infectious Diseases Unit, Internal Medicine Service, University Hospital of A Coruña, A Coruña, Spain; c Digestive Diseases Clinical Management Unit, University Hospital Virgen del Rocío, Sevilla, Spain; d Infectious Diseases, Prison Health Program, Catalan Institute of Health, Barcelona, Spain; e Liver Unit, Division of Gastroenterology & Hepatology, Hospital Universitario Central de Asturias, ISPA, Oviedo, Spain; f Gastroenterology Department, University Hospital of the Canary Islands, Spain; g Servicio de Aparato Digestivo, Hospital Clínico Universitario Lozano Blesa Zaragoza, Spain IIS Aragón, Zaragoza, Spain.

**Keywords:** aspartate aminotransferase-to-platelet ratio index, chronic hepatitis C, fibrosis-4 (FIB-4), liver cirrhosis, liver fibrosis

## Abstract

Identification of advanced fibrosis/cirrhosis in hepatitis C virus (HCV)-infected patients should be a mainstay before starting treatment; however, the limited access of many centres to transient elastography (TE) is often a barrier for early assessments. We aimed to investigate the diagnostic accuracy of serum indexes for predicting liver stiffness.

Retrospective analysis of HCV patients (with or without HIV coinfection) routinely assessed in 7 centres in Spain. The diagnostic accuracy of aspartate aminotransferase-to-platelet ratio index (APRI), fibrosis-4 (FIB-4), and their combinations was evaluated using a recent TE examination as a reference test (liver stiffness ≥ 9.5 kPa and ≥12.5 kPa for advanced fibrosis and cirrhosis, respectively). In addition to area under the receiving operating characteristic curves, sensitivity, specificity, and negative predictive value (NPV) and positive predictive value were estimated.

The analysis included 1391 patients: 346 (25%) HIV-positive, 732 (53%) people who inject drugs, and 178 (13%) incarcerated. Advanced fibrosis and cirrhosis were found in 557 (40%) and 351 (25%) patients, respectively. APRI < 0.5 (n = 595; 43%) had an NPV of 95% for excluding cirrhosis. Combined FIB-4 < 1.45 with APRI < 0.5 (n = 467; 34%) had an NPV of 87% for excluding advanced fibrosis. Combined APRI > 2 and FIB-4 > 3.25 (n = 134; 10%) had a positive predictive value of 89% for advanced fibrosis. Globally, this approach would avoid the need for TE in 53% of patients. HIV coinfection did not influence diagnostic accuracy.

Inexpensive and simple serum indexes confidently allowed identifying the absence of cirrhosis and the presence of advanced fibrosis in 53% of a heterogeneous series of real-world HCV patients with or without HIV infection.

## 1 Introduction

The advent of last-generation, direct-acting antivirals for hepatitis C virus (HCV) has increased the sustained virological rate to more than 90%.^[1]^ However, patients with cirrhosis or advanced fibrosis at the time of starting antiviral therapy remain at risk of HCV-related complications despite achieving a sustained virological response and require post-treatment surveillance for hepatocellular carcinoma.^[2]^ The need for close monitoring in these patients makes pretreatment assessment of liver fibrosis a mainstay for success, and some studies have gone in this line.^[3,4]^ Furthermore, current guidelines lack standardised criteria for diagnosing advanced fibrosis in the context of sustained virological response following treatment.

Traditionally, liver disease severity (including the stage of fibrosis) was assessed by liver biopsy, which allowed classifying liver fibrosis based on histopathological findings. However, clinical guidelines currently support noninvasive methods to assess liver disease severity before treatment.^[2,5]^ Among these, the determination of liver stiffness (LS) by transient elastography (TE) (ie, FibroScan) has gained popularity, being considered an equivalent technique for fibrosis assessment.^[6,7]^ Other noninvasive methods developed in recent years for predicting the stage of fibrosis include FibroTest and acoustic radiation force impulse elastography,^[8,9]^ or platelet count.^[10]^

Despite the good performance of commercial, noninvasive methods for assessing liver fibrosis have shown a diagnostic accuracy similar to liver biopsy, their high costs and reduced availability might be a barrier for their systematic use in HCV-infected patients. For this reason, a substantial effort is being made to investigate biomarkers that can effectively establish the degree of fibrosis in a simpler way. The aspartate aminotransferase-to-platelet ratio index (APRI) and fibrosis-4 (FIB-4) are among the serum biomarkers proposed as alternative candidates to assess the degree of fibrosis and currently account for a significant bulk of published data.^[11,12]^ However, most studies used to establish the cutoff values for these biomarkers included a low proportion of patients with mild or no fibrosis, excluded those coinfected with HIV and or HBV, and did not report data on people who inject drugs (PWID), thus losing sight of their external validity in the heterogeneous scenario often encountered in the real world. Furthermore, rather than TE, liver biopsy was typically the reference test in these studies, being increasingly used in routine practice in most centres.

Regardless of patient profiles included in previous studies, current evidence on using these 2 biomarkers separately suggests that they perform well in the upper and lower range of liver damage (ie, cirrhosis and no fibrosis). However, diagnostic accuracy drops in intermediate stages.^[13]^ Combining 2 or more methods for assessing liver fibrosis increases diagnostic accuracy^[14,15]^; however, most of these include commercial methods (eg, TE, FibroMeter, FibroTest) aside from blood parameters obtained in routine analyses allow estimating APRI and FIB-4 indices. In this study, we investigated APRI performance, FIB-4, and their combination for discriminating advanced fibrosis and cirrhosis in a large and heterogeneous retrospective series of real-world HCV-infected patients.

## 2 Materials and methods

### 2.1 Study design and patients

This was a cross-sectional study with retrospective data collection, including patients diagnosed with chronic hepatitis C in 6 hospitals and the Prison Health Program of Catalonia (Spain). Patients were consecutively recruited between January 2017 and December 2018 during a clinical visit routinely scheduled for a pretreatment assessment before starting direct-acting antiviral therapy. The diagnosis of chronic hepatitis C was established by the presence of the anti-HCV antibody and HCV RNA. Concomitant infection with HIV, confirmed by PCR, was allowed. To be included in the analysis, patients must have a medical record with TE and routine blood tests available, both performed within 3 months before enrollment.

### 2.2 Ethics statement

All data, including demographic characteristics and results of blood tests and TE examination, were retrospectively retrieved from the patient's medical record. The study was following international ethical recommendations (Helsinki Declaration and Oviedo Convention), the Spanish Government good clinical practice recommendations (Royal Decree 711/2002), and the legislation currently in force (Spanish Agency of Medicines and Health Products, Circular 15/2002). Likewise, confidentiality was ensured in compliance with the 1999 Spanish legislation regarding protecting personal data and the General Data Protection Regulation 2016/679 on data protection and privacy for all individuals within the European Union.

### 2.3 Laboratory measurements and assessment criteria

Serum HCV RNA and anti-HCV for HCV characterisation were measured using the Cobas 6800 HCV Test (Roche Diagnostics, Indianapolis, IN) and Architect (Abbot Abbott Laboratories, IL), respectively. The blood parameters needed for APRI and FIB-4 indices (ie, AST, ALT, and platelet count) were determined from a blood sample for routine assessment, obtained after 8 hours of overnight fasting. Serum indexes were computed as described elsewhere,^[16,17]^ as APRI = [{AST (IU/l)/AST_ULN (IU/l)} × 100]/platelet count (10^9^/L), and FiB-4 = [age (y) × AST (IU/L)]/[platelet count (10^9^/L) × ALT (IU/L) 1/2].

LS was measured by TE using an Echosens Fibroscan 430 mini and 630 experts. An experienced operator took 10 different measurements on the right liver lobe; the mean of 10 valid measures (ie, the interquartile range [IQR] did not exceed 30% of the median value and the success rate was 60% or greater) was used to rate the degree of fibrosis. LS thresholds of ≥12.5 kPa and ≥9.5 kPa were considered for cirrhosis (F4) and advanced fibrosis (F3–F4), respectively.^[18]^

### 2.4 Analysis

Categorical variables were described as frequencies and percentages, whereas quantitative variables were described as the mean and standard deviation and the median and IQR (defined by percentiles 25 and 75). The predictive accuracy of APRI and FIB-4 indexes were assessed by receiving operating characteristic (ROC) curve analysis, using TE as a reference test for LS. Results were reported as the mean area under the ROC (AUROC) and its 95% confidence interval (CI). Sensitivity, specificity, positive predictive value (PPV), and negative predictive value (NPV) of the serum biomarkers in predicting advanced fibrosis and cirrhosis were calculated at the following cutoff points based on the values described in the literature^[2,17]^: <0.5, <1 for F3, and >2 for F4 for APRI, and <1.45 and >3.25 for F4 for FIB-4. The corresponding parameters were also estimated to combine the 2 biomarkers at the following cutoff values: APRI <0.5 + FIB-4 < 1.45, and APRI >2 + FIB-4 > 3.25. All analyses were performed using SPSS Statistics for Windows (IBM Corp., released in 2017, Version 25.0, Armonk, NY).

## 3 Results

### 3.1 Patient characteristics

The analysis included 1391 patients: 1213 (87%) recruited from 6 participating hospitals and 178 (13%) from a prison healthcare centre. Table 1 summarises the overall study sample's main demographic and clinical characteristics and each subgroup of HIV infection status. All 346 HIV-positive patients had a median (IQR) CD4 cell count of 504 cells/μL (336–722); 97 (28%) of them were at centers for disease control and prevention stage C, and 324 (94%) had HIV RNA levels <50 copies/mL. The HIV-positive subgroup showed a higher prevalence of PWID, incarcerated patients, previously treated patients, and coinfection with hepatitis B virus. The distribution of HCV genotypes was also significantly different between both HIV groups, with HIV-positive patients showing a higher prevalence of genotype 4 and a lower prevalence of genotype 1b. No differences between groups were found regarding serum levels of HCV RNA.

**Table 1:** Demographic and clinical characteristics of study participants, grouped according to coinfection with HIV.

	Overall (n = 1391)	HCV infection (n = 1045)	HIV coinfection (n = 346)	*P*
Age (yr), *median (IQR)*	51 (45–57)	52 (45–60)	49 (45–53)	<.0001
Sex (male), *n (%)*	1019 (73)	724 (69)	295 (85)	<.0001
Alcohol intake (>50 g/d), *n (%)*	199 (17)	148 (16)	51 (18)	.30
Body mass index (kg/m^2^), *median (IQR)*	25 (22–28)	26 (23–28)	24 (22–27)	<.0001
PWID, *n (%)*	732 (53)	443 (42)	289 (84)	<.0001
Incarcerated, *n (%)*	178 (13)	110 (10)	68 (19)	.008
HCV genotype, *n (%)*				
1a	448 (32.1)	309 (29.6)	138 (39.9)	<.0001
1b	420 (30.2)	360 (34.4)	60 (17.3)	
1-other	44 (3.2)	41 (3.9)	3 (0.9)	
2	48 (3.5)	39 (3.7)	9 (2.6)	
3	221 (15.9)	167 (16)	54 (15.6)	
4	208 (15)	127 (12.2)	81 (23.4)	
Mixed	3 (0.2)	2 (0.2)	1 (0.3)	
HBsAg, *n (%)*	29 (2.1)	17 (1.6)	12 (3.5)	.030
HCV RNA (logIU/mL), *median (IQR)*	6.20 (5.70–6.60)	6.27 (5.74–6.66)	6.15 (5.68–6.64)	.39
Previous HCV therapy, *n (%)*	355 (25)	247 (23)	108 (31.2)	.005

HBsAg = hepatitis B antigen, HCV = hepatitis C virus, HIV = human immunodeficiency virus, IQR = interquartile range, PWID = people who inject drugs.

### 3.2 Test results

Median (IQR) time-lapse between blood tests and TE examinations in the overall study sample was 0 (−4; +4) days: 0 (−5; +4) and 0 (0; 2) for HIV-negative and HIV-positive patients, respectively (*P* = .03). Table 2 summarises TE results and the determination of APRI and FIB-4 scores for the overall study sample and each subgroup of HIV infection status. HIV-positive patients had significantly higher values of LS according to TE examinations, which also resulted in a greater prevalence of patients with advanced fibrosis (≥9.5 kPa) and cirrhosis (≥12.5 kPa). APRI and FIB-4 index scores were also significantly higher among HIV-positive patients.

**Table 2:** Results of the liver stiffness, assessed by transient elastography, and the serum indexes in the study sample and each of the HIV subgroups.

	Overall (n = 1391)	HCV infection (n = 1045)	HIV coinfection (n = 346)	*P*
Liver stiffness (kPa), *median (IQR)*	7.9 (5.9–12.6)	7.8 (5.7–11.9)	8.8 (6.8–14.9)	<.0001
Advanced fibrosis (≥9.5 kPa), *n (%)*	557 (40)	394 (38)	163 (47)	.002
Cirrhosis (≥12.5 kPa), *n (%)*	351 (25)	244 (23)	107 (31)	.005
APRI, *median (IQR)*	0.5 (0.3–1.1)	0.5 (0.3–0.9)	0.7 (0.4–1.4)	<.0001
FIB-4, *median (IQR)*	1.6 (1.1–2.7)	1.5 (1.04–2.5)	1.9 (1.3–3.3)	<.0001

APRI = aspartate aminotransferase to platelet ratio index, FIB-4 = fibrosis-4 index, HCV = hepatitis C virus, HIV = human immunodeficiency virus, IQR = interquartile range.

### 3.3 Diagnostic accuracy

**Figure 1:**
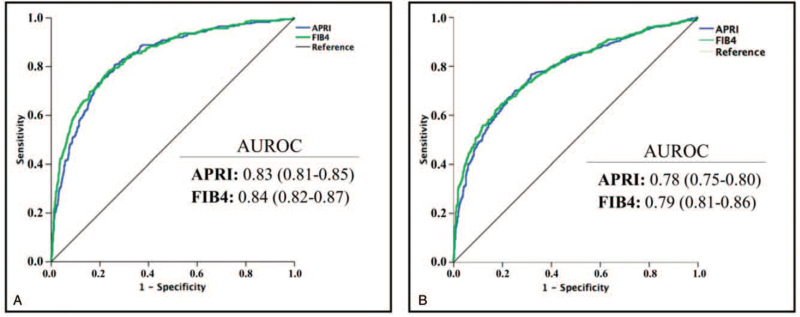
Ability of APRI and FIB-4 to predict cirrhosis **(A)** and advanced fibrosis **(B)**. The AUROC for each index is shown as median (95% confidence interval). APRI = aspartate aminotransferase to platelet ration index, AUROC = area under the receiving operating curve, FIB-4 = fibrosis-4 index.

When considered separately, APRI and FIB-4 showed high and very similar accuracy for predicting cirrhosis (Fig. 1A) and advanced fibrosis (Fig. 1B). Diagnostic parameters for each of the established cutoff points and both conditions (ie, advanced fibrosis and cirrhosis) are shown in Table 3.

**Table 3:** Performance of APRI and FIB-4 indices in discriminating advanced fibrosis and cirrhosis at various cutoff points.

		Discriminating advanced fibrosis (F3–F4)				Discriminating cirrhosis (F4)			
	Cases no. (%)	Se (%)	Sp (%)	NPV (%)	PPV (%)	Se (%)	Sp (%)	NPV (%)	PPV (%)
APRI									
<0.5	595 (43)	80	58	85	56	91	54	95	40
<1	1015 (73)	50	88	72	74	63	85	87	59
>2	150 (11)	23	97	65	87	31	96	80	73
FIB-4									
<1	292 (21)	–	–	–	–	92	27	96	31
<1.45	292 (21)	81	57	82	56	90	53	94	39
>3.25	263 (19)	40	95	70	85	53	92	85	71
APRI < 0.5 + FIB-4 < 1.45	467 (34)	86	47	84	52	94	43	95	36
APRI > 2 + FIB-4 > 3.25	134 (10)	22	98	65	90	30	97	80	78

APRI = aspartate aminotransferase to platelet ratio index, FIB-4 = fibrosis-4 index, NPV = negative predictive value, PPV = positive predictive value, Se = sensitivity, Sp = specificity.

The combination of the 2 biomarkers at a lower cutoff range (ie, APRI < 0.5 + FIB-4 < 1.45) showed the same NPV as APRI alone for discriminating cirrhosis (ie, 95% for F4 using either the combination or APRI alone). According to the values obtained, APRI alone at a cutoff of <0.5 would allow to rule out cirrhosis without the need for TE examination in 595 (43%) patients with an NPV of 95%, while APRI < 0.5 and FIB-4 < 1.45 would suggest the absence of advanced fibrosis with an NPV of 84%. The upper cutoff range of the combination (ie, APRI > 2 and FIB-4 > 3.25) showed a higher PPV than the indices alone for discriminating advanced fibrosis. In the study cohort, this combined cutoff would allow confirming advanced fibrosis (ie, ≥F3) without the need for TE examination in 134 (10%) patients with a PPV of 90%. Globally, the use of APRI <0.5 and the combination of APRI > 2 and FIB-4 > 3.25 would allow discriminating the presence/absence of advanced fibrosis without the need for TE examination in 729 (53%) patients in the study cohort.

Of the 595 patients with APRI < 0.5, 110 (32% of the global cohort) were HIV-positive. NPV in these patients was similar to that of HCV mono-infected ones (94% vs 95%) (Table 4). Conversely, PPV of the combined index (ie, APRI > 2 + FIB-4 > 3.25) for advanced fibrosis decreased in HIV-negative patients, accounting for 14% (n = 48) of patients in the cohort, being 93% and 85% for HIV-negative and HIV-positive patients, respectively.

**Table 4:** Performance of APRI and FIB-4 indices in discriminating advanced fibrosis and cirrhosis according to the HIV infection status.

	Cases no. (%)	Se (%)	Sp (%)	NPV (%)	PPV (%)
APRI < 0.5 for F4					
HIV positive	110 (32)	93	43	94	42
HIV negative	485 (46)	90	57	95	39
FIB-4 < 1.45 for F3–F4					
HIV positive	106 (31)	86	45	78	58
HIV negative	476 (46)	79	60	83	55
APRI > 2 + FIB-4 > for F3–F4					
HIV positive	48 (14)	25	96	60	85
HIV negative	86 (8)	20	99	67	93

APRI = aspartate aminotransferase to platelet ratio index, FIB-4 = fibrosis-4 index, HIV = human immunodeficiency virus, NPV = negative predictive value, PPV = positive predictive value, Se = sensitivity, Sp = specificity.

## 4 Discussion

In this large retrospective series of HCV patients, including those coinfected with HIV, the use of APRI at a cutoff level of 0.5 showed an NPV of 95% for cirrhosis, irrespective of HIV coinfection, and the combined index of APRI < 0.5 + FIB-4 < 1.45 suggested absence of advanced fibrosis. The 20% cirrhosis rate observed in our cohort is in line with other observational studies in which APRI and FIB-4 were used as noninvasive tests to gather epidemiological information of the fibrosis stage.^[19,20]^ The combination of APRI > 2 and FIB-4 > 3.25 increased the diagnostic accuracy for advanced fibrosis compared with the indices alone, reaching a PPV of 90% for ≥F3. The accuracy in excluding advanced fibrosis remained unchanged in HCV/HIV coinfected patients, although PPV slightly decreased in these patients.

Of the 2 indices included in the combination, APRI has been more extensively investigated. Although all studies have reported high accuracy of this index for predicting advanced fibrosis and cirrhosis, meta-analyses indicate remarkably heterogeneous results.^[11,12]^ The median AUROC observed in our cohort for predicting cirrhosis, which was based on the APRI score (0.83; 95% CI 0.81–0.85), was similar to that reported in a pooled analysis including 16,694 HCV-positive patients for predicting cirrhosis (0.84; 95% CI 0.54–0.97) and advanced fibrosis (0.78 vs 0.77; 95% CI 0.58–0.95).^[11]^ The accuracy of FIB-4, less frequently reported, followed a similar trend, with median AUROC values for cirrhosis and advanced fibrosis in line with those obtained from the pooled analysis, which reported median AUROC values of 0.74 (0.61–0.81) for advanced fibrosis and 0.87 (0.83–0.92) for cirrhosis.

The combination of both indices added limited predictive value to APRI alone for excluding cirrhosis (NPV remained at 95% and specificity decreased from 54%–43%) and advanced fibrosis (NPV increased from 82%–84% and specificity decreased from 58%–47%). A low NPV for advanced fibrosis indicates that even the combination of both indices should be considered only suggestive of ≥F3 and that further exams must be conducted to establish the degree of fibrosis. Conversely, the accuracy in confirming the presence of advanced fibrosis was remarkably improved when adding FIB-4 > 3.25 to APRI > 2, resulting in a PPV of 90%, 3% to 5% points greater than the individual PPV for advanced fibrosis (87% and 85% for APRI and FIB-4, respectively). To our knowledge, this approach of combining APRI and FIB-4 without adding any other test has been barely investigated. Crisan et al^[15]^ explored this combination in a cohort of 446 HCV-mono-infected patients and found a PPV of 83% for ≥F3 using liver biopsy as a reference test. Although the different clinical characteristics of our cohort may contribute to this divergence, the use of liver biopsy instead of TE for grading fibrosis likely plays an essential role in the differences observed.

According to the results obtained, fibrosis could be graded with high accuracy in 53% of cases without TE examination. The rest of the patients (ie, APRI between 0.5 and 2, and FIB-4 ≤ 3.25) would remain at high risk of advanced fibrosis with a limited capacity of the biomarkers to confirm it. This lower performance in discriminating intermediate stages has also been observed when using other noninvasive methods. Thus, TE has high accuracy in ruling out advanced fibrosis and cirrhosis but limited capacity to classify patients between F3 and F4.^[9,21,22]^ Compared with TE, biomarkers have higher reliability and a much lower cost.^[23]^ Hence, these biomarkers may provide healthcare centres with a nonexpensive and reliable tool for screening advanced fibrosis, saving time and reducing referral rates to tertiary hospitals. This advantage is particularly relevant for remote care settings (eg, telemedicine-based) such as drug addiction centres, for which biomarkers may be a valuable alternative to TE.^[24]^

Ease of access to diagnostic methods for classifying fibrosis is of particular interest for outpatient centres, including those managing a significant proportion of high-risk patients, such as prison healthcare sites. However, these patients are rarely involved in studies investigating the diagnostic accuracy of biomarkers. Our cohort included a remarkable number of PWID (53%) and HIV/HCV coinfected patients (25%). Although it is recommended that all these populations follow the same therapeutic approach and monitoring as HCV-mono-infected patients, some authors have reported a lower accuracy of APRI for the identification of severe fibrosis and cirrhosis in coinfected patients, partially explained by the higher risk of thrombopenia associated with HIV infection.^[25]^ In our analysis, NPV for advanced fibrosis was similar in HIV-positive and HIV-negative patients (94% and 95%, respectively). However, PPV dropped from 93% in HIV-negative patients to 85% in HIV-positive patients. Consistently with previous studies,^[26]^ our findings confirm the predictive capacity of APRI at the cutoff of 0.5 for ruling out cirrhosis in HIV/HCV coinfected patients, although caution is suggested when predicting the presence of advanced fibrosis in these patients.

The development of highly effective pangenotypic and panfibrotic drugs has promoted simplification strategies in the serological/virological diagnosis of hepatitis C through a single-step diagnosis. Along the same lines, our study shows the usefulness in real life of the serological tests (FIB-4 and APRI) that allow us to diagnose advanced fibrosis or exclude the presence of cirrhosis in more than 50% of HCV-monoinfected or HCV/HIV-coinfected patients, who are going to start treatment, thus, facilitating the diagnosis of the degree of fibrosis and being able to start treatment in the same consultation. At the current COVID pandemic, these results acquire more relevance in the treatment and eradication strategies of hepatitis C.

One of the study limitations could be that the results obtained to classify the degree of fibrosis in patients have not been compared with those obtained from a liver biopsy, considered the standard gold test. This approach could limit the precision of the assessed parameters in terms of absolute diagnostic performance. However, it is worth highlighting the increasingly widespread recommendation of noninvasive tests as diagnostic methods. This fact has favoured the implementation of TE as the reference test in many tertiary hospitals of our country, but not affordable by all hospitals or medical centres. This fact makes it challenging to have a large sample of patients diagnosed by liver biopsy, but at the same time, it contributes to the justification of the objective of our study. Our primary interest was investigating the circumstances in which serum biomarkers could be comparable to those obtained by TE to classify fibrosis. Another limitation would be that the study was only to patients with active HCV infection alone or with HIV coinfection, which would justify future studies investigating the performance of these biomarkers in patients with a sustained virological response and would not be applicable to other liver viruses (ie, VHB) that can cause liver fibrosis.

In summary, our results show that APRI and FIB-4 indices, obtained from serum parameters routinely analysed, allowed identifying the absence of cirrhosis and the presence of advanced fibrosis in nearly half the patients in a real-life series of HCV with or without concomitant HIV infection. These findings provide clinicians with confident tools to avoid TE examination – often not available in the visiting centre – in a remarkable number of HCV-infected patients, considering the large sample size and the representativity of our cohort. This approach may reduce the management cost of these patients and shorten the time lapse between the diagnosis and grading of fibrosis and treatment start.

## Acknowledgments

The authors would like to thank Gerard Carot-Sans, PhD, for providing medical writing assistance during the manuscript preparation and revision on behalf of BioClever 2005, SLU.

## Author contributions

**Conceptualization:** Nicolás Merchante, Álvaro Mena, Juan-Manuel Pascasio, Andrés Marco, Manuel Rodriguez, Manuel Hernandez-Guerra, Miguel-Angel Simón.

**Data curation:** Nicolás Merchante, Álvaro Mena, Juan-Manuel Pascasio, Andrés Marco, Manuel Rodriguez, Manuel Hernandez-Guerra, Miguel-Angel Simón.

**Formal analysis:** Nicolás Merchante, Miguel-Angel Simón.

**Funding acquisition:** Nicolás Merchante, Miguel-Angel Simón.

**Investigation:** Nicolás Merchante, Álvaro Mena, Juan-Manuel Pascasio, Andrés Marco, Manuel Rodriguez, Manuel Hernandez-Guerra, Miguel-Angel Simón.

**Methodology:** Nicolás Merchante, Álvaro Mena, Juan-Manuel Pascasio, Manuel Rodriguez, Manuel Hernandez-Guerra, Miguel-Angel Simón.

**Project administration:** Nicolás Merchante, Miguel-Angel Simón.

**Resources:** Nicolás Merchante, Miguel-Angel Simón.

**Software:** Nicolás Merchante, Miguel-Angel Simón.

**Supervision:** Nicolás Merchante, Miguel-Angel Simón.

**Validation:** Nicolás Merchante, Álvaro Mena, Juan-Manuel Pascasio, Andrés Marco, Manuel Rodriguez, Miguel-Angel Simón.

**Visualization:** Nicolás Merchante, Álvaro Mena, Juan-Manuel Pascasio, Andrés Marco, Manuel Rodriguez, Manuel Hernandez-Guerra, Miguel-Angel Simón.

**Writing – original draft:** Nicolás Merchante, Miguel-Angel Simón.

**Writing – review & editing:** Nicolás Merchante, Álvaro Mena, Juan-Manuel Pascasio, Andrés Marco, Manuel Rodriguez, Manuel Hernandez-Guerra, Miguel-Angel Simón.
